# Oral Rg1 supplementation strengthens antioxidant defense system against exercise-induced oxidative stress in rat skeletal muscles

**DOI:** 10.1186/1550-2783-9-23

**Published:** 2012-05-18

**Authors:** Szu-Hsien Yu, Hui-Yu Huang, Mallikarjuna Korivi, Ming-Fen Hsu, Chih-Yang Huang, Chien-Wen Hou, Chung-Yu Chen, Chung-Lan Kao, Ru-Ping Lee, Shin-Da Lee, Chia-Hua Kuo

**Affiliations:** 1 Laboratory of Exercise Biochemistry, Taipei Physical Education College, Taipei City, Taiwan; 2 Department of Food Science, Nutrition, and Nutraceutical Biotechnology, Shih-Chien University, Taipei City, 10462, Taiwan; 3 Graduate Institute of Basic Medical Science, China Medical University, Taichung, Taiwan; 4 Department of Health and Nutrition Biotechnology, Asia University, Taichung, Taiwan; 5 Department of Physical Therapy and Graduate Institute of Rehabilitation Science, China Medical University, Taichung, Taiwan; 6 Department of Healthcare Administration, Asia University, Taichung, Taiwan; 7 Department of Physical Medicine and Rehabilitation, Taipei Veterans General Hospital, National Yang-Ming University, Taipei, Taiwan; 8 Department of Nursing, Tzu Chi University, Hualien, Taiwan

**Keywords:** Ginseng, Ginsenoside, Antioxidant status, Lipid peroxidation, Swimming, MDA, Protein carbonyl, Oxidative damage, Free radical attack, Sports nutrition, Sarcolemma

## Abstract

**Background:**

Previous studies reported divergent results on nutraceutical actions and free radical scavenging capability of ginseng extracts. Variations in ginsenoside profile of ginseng due to different soil and cultivating season may contribute to the inconsistency. To circumvent this drawback, we assessed the effect of major ginsenoside-Rg1 (Rg1) on skeletal muscle antioxidant defense system against exhaustive exercise-induced oxidative stress.

**Methods:**

Forty weight-matched rats were evenly divided into control (N = 20) and Rg1 (N = 20) groups. Rg1 was orally administered at the dose of 0.1 mg/kg bodyweight per day for 10-week. After this long-term Rg1 administration, ten rats from each group performed an exhaustive swimming, and remaining rats considered as non-exercise control. Tibialis anterior (TA) muscles were surgically collected immediately after exercise along with non-exercise rats.

**Results:**

Exhaustive exercise significantly (p<0.05) increased the lipid peroxidation of control group, as evidenced by elevated malondialdehyde (MDA) levels. The increased oxidative stress after exercise was also confirmed by decreased reduced glutathione to oxidized glutathione ratio (GSH/GSSG ratio) in control rats. However, these changes were completely eliminated in Rg1 group. Catalase (CAT) and glutathione peroxidase (GPx) activities were significantly (p<0.05) increased by Rg1 in non-exercise rats, while no significant change after exercise. Nevertheless, glutathione reductase (GR) and glutathione S-transferase (GST) activities were significantly increased after exercise in Rg1 group.

**Conclusions:**

This study provide compelling evidences that Rg1 supplementation can strengthen antioxidant defense system in skeletal muscle and completely attenuate the membrane lipid peroxidation induced by exhaustive exercise. Our findings suggest that Rg1 can use as a nutraceutical supplement to buffer the exhaustive exercise-induced oxidative stress.

## Introduction

Disruption in the balance between free radical production and scavenging capability contributes to the accumulation of oxidative damage in muscle tissues. Under normal physiological conditions this balance is tightly buffered by the antioxidant enzymes, which consists of superoxide dismutase (SOD), catalase (CAT), and glutathione peroxidase (GPx), as well as non-enzymatic antioxidant reduced glutathione (GSH) [1,2]. Moderate exercise training has been shown to improve this antioxidant defense system to maintain the stable redox status against the recurrence of exercise-induced oxidative stress [3]. However, acute exhaustive exercise impairs the system due to overwhelming production of reactive oxygen species (ROS) in skeletal muscle [2]. As a result, accumulated excessive ROS can attack the vital biomolecules, such as plasma membrane lipids and proteins, and therefore deteriorates normal cellular functions. This scenario has been well documented by observation of elevated lipid peroxidation (malondialdehyde, MDA) and protein carbonyl (PC) after exhaustive exercise in different tissues of rat [4-6]. Preservation of cellular integrity for normal recovery by nutraceutical products against oxidative stress during high level sports competition represents a market demand for athletes during competition season.

*Panax ginseng* extracts have been shown to up-regulate the antioxidant defense system and attenuate the oxidative stress in rats [7,8]. However, nutraceutical actions of ginseng extracts have been controversial in many studies [9,10]. Ginsenosides, a class of steroidal glycosides, are considered as the main bioactive components in *P. ginseng* that are thought to be responsible for the nutraceutical actions. The ginsenoside constituents in *P. ginseng* can be varied by season, cultivating soil and extraction processes [11,12]. Some ginsenosides have different or even opposing pharmacological actions than others on free radical scavenging capacity [9,10]. Among various ginsenosides (protopanaxadiols: Rb1, Rb2, Rc, Rd and protopanaxatriols: Rg1, Re, Rf), ginsenoside-Rg1 (Rg1) is one of the major compound in *P. ginseng*[13].

It is currently unknown whether prolonged pre-administration of Rg1 can protect the skeletal muscle against exhaustive exercise-induced oxidative stress. Available evidences have shown that Rg1 is able to attenuate oxidative damage against ischemic reperfusion and dopamine-induced damage in rat tissues [14,15]. Thus, we hypothesized that exhaustive exercise-induced oxidative damage in rat skeletal muscle can be prevented by Rg1 pretreatment. Oxidative damage markers, enzymatic and non-enzymatic antioxidant defense system were determined in rat skeletal muscle.

## Methods

### Animal care and maintenance

Forty male Sprague Dawley (SD) rats, weighting 410 ± 10 g (4-month old) were obtained from the LASCO (Taipei, Taiwan) and used for this study. All the animals were housed under temperature (22 ± 2°C) and relative humidity (55%) controlled room with 12/12 h light/dark cycle. Two rats in each cage were maintained. All rats were fed standard laboratory chow (PMI Nutrition International, Brentwood, MO, USA) and water *ad libitum*. This study was approved by the Animal Care and Use Committee of Taipei Physical Education College, and conformed to the Guidelines for the Use of Research Animals published by the Council of Agriculture, Executive Yuan, Taiwan.

### Plant extract and chemicals

**Figure 1:**
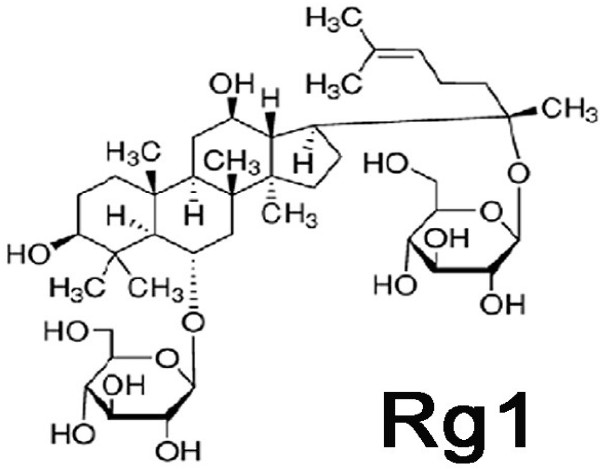
Chemical structure of ginsenoside-Rg1.

Ginsenodie-Rg1 (Rg1, molecular weight 801.01, Figure 1) was obtained from the NuLiv Science USA, Inc, Walnut, CA, USA. All the other chemicals used in this study were obtained from Sigma Chemicals (St. Louis, MO, USA) and Cayman Chemical Company (Ann Arbor, MI, USA).

### Grouping and treatment

Weight matched rats were equally divided into control (N = 20) and Rg1 (N = 20) groups. Rg1 was dissolved in 0.9% saline, and administered to Rg1 group daily at the dose of 0.1 mg/kg body weight (b.w) by gastric gavage for 10 weeks. Similarly, control group rats received the same amount of saline for the same duration.

### Exercise protocol

In this study, rats performed swimming until exhaustion in a water pool. The water temperature was maintained at 33 ± 1°C. Three days prior to acute exhaustive swimming challenge, all animals were familiarized with swimming environment for 10 min/day. Then, half number of rats (N = 10) from each group were performed an exhaustive swimming with a lead ingot (3% body weight) loaded to the tail of each rat. Rats were swimming until exhaustion and clearly monitored to avoid sink in the pool. The swimming duration was not significantly different between control and Rg1 groups.

### Tissue collection

Immediately after exhaustive exercise, rats were anesthetized with chloral hydrate injection (400 mg/kg b.w., intraperitoneally). The tibialis anterior (TA) muscle from the hind limbs of exercised and non-exercised rats were quickly excised and frozen into liquid nitrogen, and then stored at −80°C until biochemical analyses. 100 mg of muscle tissue was homogenized in 1 mL of Tris buffer (50 mM, pH 7.5) and centrifuged at 10000 g for 10 min at 4°C. Collected supernatant was used for the estimation of protein carbonyl (PC) and glutathione levels. The same supernatant was also used to measure the activities of catalase (CAT), glutathione peroxidase (GPx), glutathione reductase (GR), glutathione S-transferase (GST) and xanthine oxidase (XO).

### Determination of lipid and protein oxidation

Lipid peroxidation marker malondialdehyde (MDA) in muscle samples was measured spectrophotometrically as described by Ohkawa et al. [16]. Muscle tissue was homogenized in phosphate buffer (50 mM, pH 7.0) and centrifuged at 10000 g for 10 min at 4°C. This assay is based on the MDA-TBA (thiobarbituric acid) compound formed by the reaction between MDA and TBA at high temperature (90-100°C). The MDA-TBA was quantified at 450 nm by spectrophotometer.

Protein oxidation in the muscle samples was determined by measuring the protein carbonyl residues by using the DNPH (2,4-dinitrophenylhydrazine). According to the protocol provided by Cayman’s protein carbonyl assay kit (Cayman Chemical Company, Ann Arbor, MI, USA), the amount of protein-hydrozone product was quantified spectrophotometrically at wavelength of 360 nm (Tecan Genios, A-5082, Austria).

### Measurement of reduced and oxidized glutathione levels

Glutathione assay kit (Cayman Chemical Company, Ann Arbor, MI, USA) was used to measure the reduced glutathione (GSH) and oxidized glutathione (GSSG) levels in muscle. The reaction between GSH and DTNB (5,5′-dithio-*bis*-2- nitrobenzoic acid) results a colored product TNB (5-thio-2-nitrobenzoic acid). The absorbance of TNB was measured at 405 nm by ELISA plate reader (Tecan Genios, A-5082, Austria).

### Assessment of antioxidant enzyme activities

For determination of superoxide dismutase (SOD) activity, muscle samples were homogenated in 20 mM HEPES buffer (pH 7.2) containing 1 mM EGTA, 210 mM mannitol, and 70 mM sucrose. The principle of SOD assay is based on the ability of SOD to reduce superoxide radicals (O_2_^**·─**^−) generated by xanthine oxidase (XO). The absorbance of the sample was read at 450 nm using ELISA plate reader (Tecan Genios, A-5082, Austria). SOD activity was expressed as U/mg protein. Catalase (CAT) activity was measured by adding the hydrogen peroxide (H_2_O_2_) to the samples and absorbance was read at 540 nm using ELISA plate reader (Tecan Genios, A-5082, Austria). Catalase activity was expressed as nano mole formaldehyde/min/ mg protein.

Both glutathione peroxidase (GPx) and glutathione reductase (GR) enzyme activities were measured in accordance with the protocols supplied by the manufacturer. The decreased in the absorbance of oxidation of NADPH was measured at 340 nm once every minute to obtain at least 5 time points using a plate reader (Tecan Genios, A-5082, Austria). The kits from Cayman Chemical Company (Ann Arbor, MI, USA) were used to determinate all these antioxidant enzymes. Enzyme activities were calculated per mg protein.

### Measurement of xanthine oxidase activity

As a source of free radical production, xanthine oxidase (XO) activity was assayed based on the H_2_O_2_ production during oxidation of hypoxanthine. This assay was performed by the protocol provided by Cayman Chemical Company (Ann Arbor, MI, USA). Briefly, H_2_O_2_ reacts with ADPH (10-acetyl-3, 7-dihydroxyphenoxazine) in presence of HRP (horseradish peroxidase) to produce resourfin, a highly fluorescent compound, which was analyzed at 535 nm (excitation) and 585 nm (emission) using ELISA plate reader (Tecan Genios, A-5082, Austria). XO activity was expressed as mU/mg protein.

Muscle protein concentrations were determined by the Bio-Rad protein assay reagent (BioRad Laboratories, Hercules, CA, USA).

### Statistical analyses

SPSS (version 17.0) was used to analyze the data. All the values were shown as mean ± standard error (SE) for ten replicates. One-way analysis of variance (ANOVA) with Duncan *post hoc* test was used to evaluate the significant differences between both groups. *P* value was set at 0.05 and considered statistically significant.

## Results

**Figure 2:**
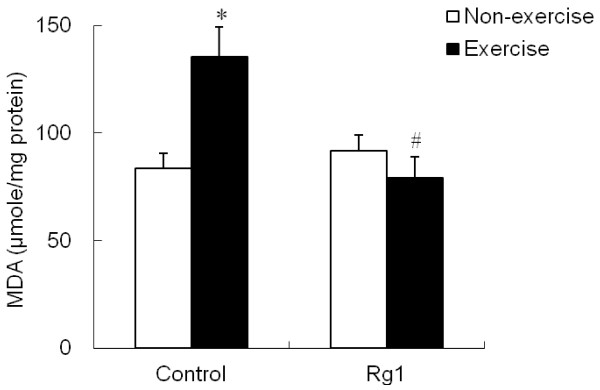
**Effect of Rg1 administration on muscle MDA levels in exhaustive exercised rats.** * indicates significant difference against control non-exercise group. # indicates significant difference against control exercise group.

**Figure 3:**
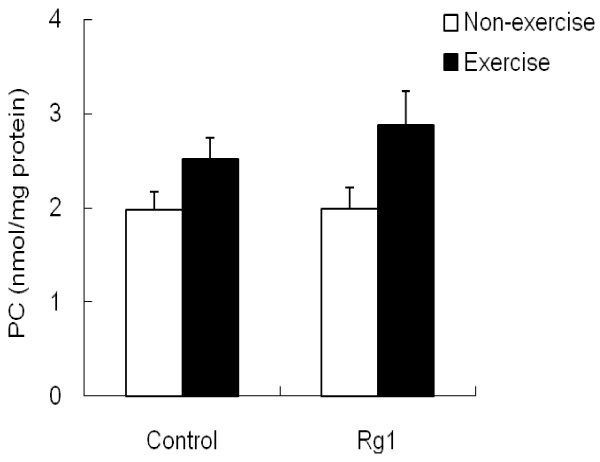
Effect of Rg1 administration on muscle PC levels in exhaustive exercised rats.

Exhaustive exercise-induced lipid peroxidation and protein oxidation in muscle tissues were estimated by measuring the levels of MDA and PC, respectively. Exhaustive swimming significantly (p <0.05) increased the MDA levels in control group, which indicates increased sacrolemma lipid peroxidation in muscle tissue. Exercise-induced elevation in MDA levels were significantly (p <0.05) attenuated in Rg1 group (Figure 2). However, no significant change in muscle protein carbonyl levels was noticed either by exhaustive exercise or by Rg1 treatment (Figure 3).

**Figure 4:**
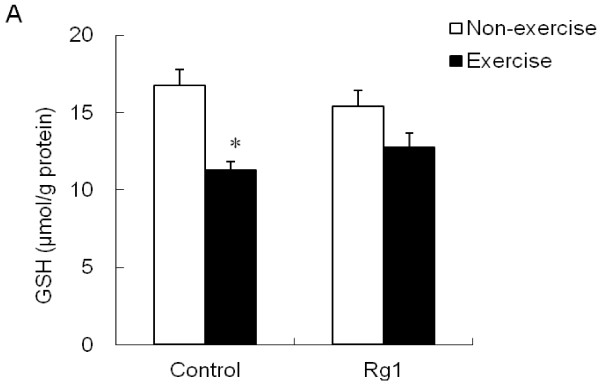
**Effect of Rg1 administration on muscle GSH levels (A) and GSH/GSSG ratio (B) in exhaustive exercised rats.** * indicates significant difference against control non-exercise group. # indicates significant difference against control exercise group.

The changes in GSH content and GSH/GSSG ratio are shown in Figure 4A and 4B. Skeletal muscle GSH content was drastically (p <0.05) decreased after exhaustive exercise in control group. However, this decrease was not found in Rg1 pretreated exercised rats. Similarly, GSH/GSSG ratio was also decreased after exercise in control group. The loss in GSH/GSSG ratio was rescued in Rg1 pretreated exercised rats, and this was significantly higher compared to control exercised rats.

**Figure 5:**
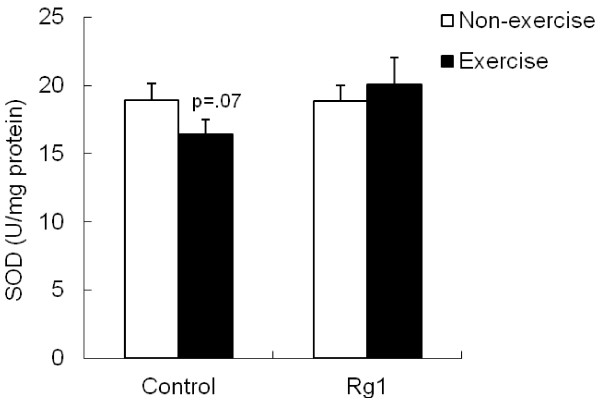
Effect of Rg1 administration on muscle SOD activity in exhaustive exercised rats.

**Figure 6:**
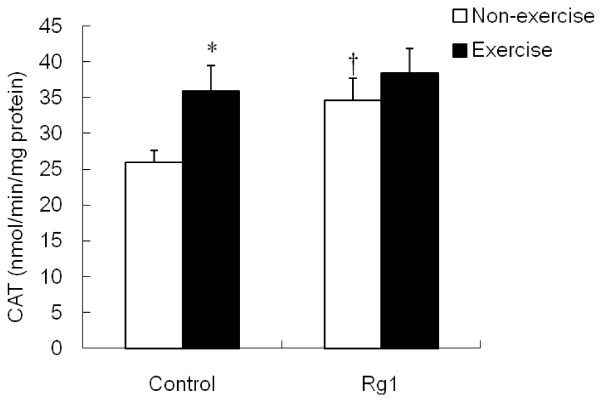
**Effect of Rg1 administration on muscle CAT activity in exhaustive exercised rats.** * indicates significant difference against control non-exercise group. † indicates significant difference against control non-exercise group.

Exhaustive exercise marginally (p <0.07) decreased SOD activity in control group (Figure 5), but this decrease was not significant in Rg1 group. In contrast to SOD results, CAT was increased significantly (p <0.05) after exhaustive exercise in control group compared to non-exercise rats (Figure 6). Rg1 treatment also increased CAT activity in non-exercise rats, while, no effect of Rg1 after exhaustive exercise.

**Figure 7:**
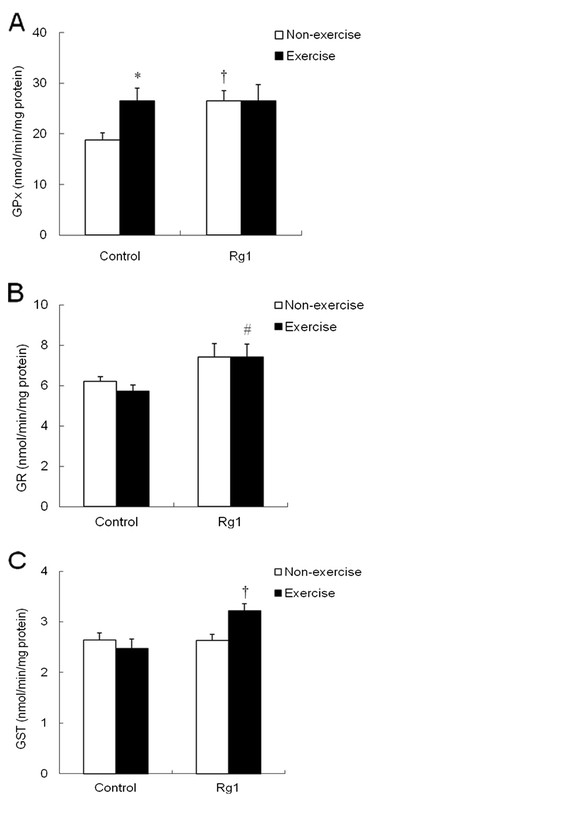
**Effect of Rg1 administration on muscle GPx (A), GR (B) and GST (C) activities in exhaustive exercised rats.** * indicates significantly different from control non-exercise group. † indicates significant difference against control non-exercise group. # indicates significant difference against control exercise group.

Exhaustive exercise significantly (p <0.05) increased the GPx activity in control group, but no change in Rg1 group (Figure 7A). Nevertheless, Rg1 alone increased the GPx activity in non-exercise rats. In contrast to GPx response, GR activity was not influenced by exhaustive exercise in control group, but increased in Rg1 group after exercise. This increase was statistically significant compared to control exercise rats (Figure 7B). Similar with GR, GST activity was also not influenced by exercise in control group, but increased after exercise in Rg1 group compared to control group (Figure 7C).

**Figure 8:**
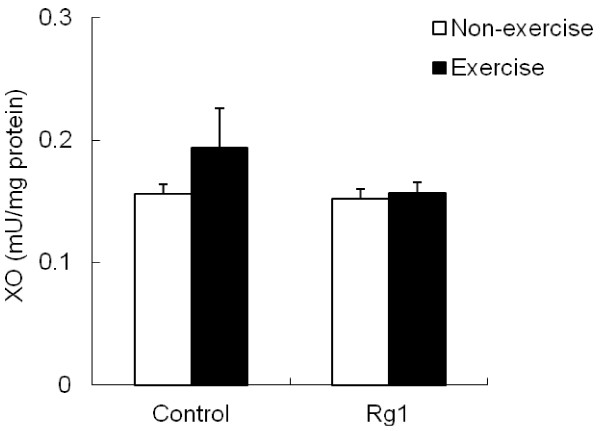
Effect of Rg1 administration on muscle XO activity in exhaustive exercised rats.

XO activity was shown in Figure 8. Muscle XO activity increased after exercise was not statistically significant (p =0.24).

## Discussion

The major finding of the study is that long-term oral Rg1 supplementation can strengthen antioxidant defense capability in skeletal muscle and attenuate the oxidative damage induced by an acute bout of exhaustive exercise. In particular, exhaustive exercise-induced membrane lipid peroxidation was effectively eliminated in the skeletal muscle of rats, which pre-treated with Rg1. In line with this finding, decreased GSH/GSSG ratio after exercise was prevented in the Rg1 group. These results provide compelling evidence that oral Rg1 supplementation can protect sarcolemma against exercise-induced oxidative stress by enhancing antioxidant system of skeletal muscle.

Minimizing of unwanted side reactions like lipid peroxidation and protein oxidation is essential in preserving normal function of cells, since all chemical reactions in human cells are under strict enzymatic regulation to conform a tightly controlled metabolic program. These are largely relying on maintaining normal structure of biomolecules against metabolic perturbation. However, increasing physical work unavoidably increases the production of O_2_^·−^ and hydroxyl radicals ^*^OH, which consequently attack the membrane lipids and results in MDA formation [2]. Ginseng extracts has been shown to decrease the MDA levels and muscle damage caused by eccentric exercise in rats [17]. As a major component of ginsenosides, Rg1 has been found to reduce the MDA levels in liver and brain of rats [18]. The present study adds to the current knowledge that Rg1 may be the key ginsenoside component, which contributes to the protective effect of ginseng against exercise-induced lipid peroxidation in skeletal muscle.

Increased MDA levels confirm the increased of oxidative stress by exhaustive exercise. However, protein carbonyls as an indicator of protein oxidation were not significantly increased after exhaustive exercise. The previous reports on protein carbonyls after exercise show mixed results. For instance, protein oxidation in human blood was elevated after resistance exercise [19]. Another study showed that plasma MDA levels were inversely correlated with protein carbonyls under betamethasone-induced oxidative stress condition [20]. The possible reason for this discrepancy may be related to the differences in experimental design and model used. Alternatively, elevated protein degradation during prolonged exercise may affect the level of protein oxidation [21].

As per our knowledge, this is the first report to demonstrate the significant effect of Rg1 on preserving GSH/GSSG ratio, in parallel with up-regulated GR activity in the skeletal muscle of exercised rats. Preserving GSH/GSSG ratio can happen by either increasing GSH biosynthesis or activating GSH-recycle enzyme (GR) activity [22]. In this study, increased GR activity in Rg1-treated exercised rats may contribute to the preservation of GSH/GSSG ratio. Red ginseng extract has been shown to elevate the rate-limiting enzyme of GSH-biosynthesis and protect the cells from oxidative cell death [23]. Furthermore, pretreatment of protopanaxatriol containing Rg1 has been reported to boost the GR activity and maintain the stable GSH/GSSG ratio against H_2_O_2_-induced oxidative stress in endothelial cells [24]. Therefore, Rg1 may be the active component of protopanaxatriol that accounts for stabilization of GSH/GSSG ratio against various types of external challenges. Furthermore, GST acts to conjugate peroxidized lipids to GSH [22]. In our study, muscle GST activity was not affected by exhaustive exercise, which agreed with the results reported by Malaguti et al. [25]. Yet, muscle GST activity was increased in Rg1 pre-treatment rats which may partly contribute to the attenuated lipid peroxidation after exercise.

Endogenous free radicals are removed by a set of antioxidant enzymes, including SOD, CAT, and GPx. Previous studies have shown increased [26], decreased [27] or no change [28] in SOD activity after exhaustive exercise. Our data showed marginally decreased SOD activity after exhaustive exercise in control group. Furthermore, CAT and GPx works in decomposing the toxic H_2_O_2_ to water and oxygen. Here, both CAT and GPx activities showed similar response after long-term Rg1 supplementation and acute exercise. Increases in CAT and GPx in exercised rats are noted as a compensatory response against excessive H_2_O_2_ levels [29,30]. However, Taysi et al. [31] reported decreased liver CAT activity after exhaustive treadmill running. This discrepancy might be due to tissue specific response or mode of exercise. Increased GPx activity was similar with the findings by Caillaud et al. [28], who reported increased muscle GPx activity after exercise. Ginseng saponins have been shown to increase CAT gene expression and protect the liver from thioacetamide-induced injury [32]. Voces et al. [33] reported improved liver antioxidant status along with GPx activity by ginseng extracts. Rg1 supplementation also increased CAT and GPx activities in non-exercise rats, which may explain, in part, the enhanced antioxidant defense system by ginseng.

## Conclusion

The results of the study provide strong evidence that long-term Rg1 supplementation can effectively attenuate the exhaustive exercise-induced increased lipid peroxidation and decreased GSH/GSSG ratio in rat skeletal muscle. The beneficial effect of Rg1 is also explained, in part, by the steady state maintenance of antioxidant defense system in the skeletal muscle. The finding of the study suggests that Rg1 can be used to design nutraceutical supplements aimed to preserve normal biomolecular structure of skeletal muscle against exhaustive exercise-induced oxidative stress, which might be important in preventing loss of cellular function and warrants quick recovery after sports competition.

## Competing interests

The authors declare that they have no competing interests.

## Authors’ contributions

All authors were responsible for the study design, data collection, statistical analysis, and preparation of the manuscript. All authors read and approved the final manuscript.
